# Ventral tegmental area activation promotes firing precision and strength through circuit inhibition in the primary auditory cortex

**DOI:** 10.3389/fncir.2014.00025

**Published:** 2014-03-20

**Authors:** Yunxiao Lou, Wenzhi Luo, Guangwei Zhang, Can Tao, Penghui Chen, Yi Zhou, Ying Xiong

**Affiliations:** ^1^Chongqing Key Laboratory of Neurobiology, Department of Neurobiology, Third Military Medical UniversityChongqing, China; ^2^Battalion Cadet Brigade 7, Third Military Medical UniversityChongqing, China

**Keywords:** VTA, auditory cortex, intracellular, *in vivo*, inhibition

## Abstract

The activation of the ventral tegmental area (VTA) can rebuild the tonotopic representation in the primary auditory cortex (A1), but the cellular mechanisms remain largely unknown. Here, we investigated the firing patterns and membrane potential dynamics of neurons in A1 under the influence of VTA activation using *in vivo* intracellular recording. We found that VTA activation can significantly reduce the variability of sound evoked responses and promote the firing precision and strength of A1 neurons. Furthermore, the compressed response window was caused by an early hyperpolarization as a result of enhanced circuit inhibition. Our study suggested a possible mechanism of how the reward system affects information processing in sensory cortex: VTA activation strengthens cortical inhibition, which shortens the response window of post-synaptic cortical neurons and further promotes the precision and strength of neuronal activity.

## INTRODUCTION

There are numerous functional and anatomical connections between the sensory system and the reward system. Psychological studies have shown increased interactions between the auditory cortex and mesolimbic reward circuitry during esthetic perceptual tasks (Salimpoor et al., 2013). In the visual cortex, dopaminergic reward signals selectively gate cortical plasticity (Arsenault et al., 2013) and visual learning is mediated by stimulus-reward pairing in the absence of attention (Seitz et al., 2009). In the auditory system, dopaminergic inputs are required for sound sequence discrimination learning (Kudoh and Shibuki, 2006) and could modulate the consolidation of memory for complex sounds in the auditory cortex (Schicknick et al., 2008). In the inferior colliculus, dopamine modulates neural activity in a heterogeneous manner (Gittelman et al., 2013). During stimulus-reward associative learning, the auditory cortex shows rapid plasticity in the spectrotemporal receptive field (David et al., 2012). In humans, dopaminergic modulation also influences neural activity and induces learning-dependent plasticity in the auditory cortex (Weis et al., 2012; Puschmann et al., 2013). These reports suggest that the dopaminergic reward system may play an important role in auditory processing.

As an important part of the mesencephalic reward circuitry, the ventral tegmental area (VTA) has often been considered a potential center for distributing reward information to various sensory cortices (Tan, 2009; Kuleshova et al., 2010), and may play an important role in sensory information processing. In mature rats, tonotopic representation in the auditory cortex can still be largely rebuilt by paired activation of the VTA and auditory neurons (Bao et al., 2001, 2003). However, the cellular mechanisms for how VTA activation can modulate the activity of neurons in the sensory cortex remain largely unknown. Previous studies have suggested that VTA activation followed by specific stimuli can suppress the response to subsequent stimulation in both the visual and auditory cortex (Bao et al., 2003; Arsenault et al., 2013). It is still unclear if this modulation is due to changes in cellular excitability or caused by a circuit mechanism because it has been reported that either mechanism could contribute to the alteration (Wu et al., 2008; Favero et al., 2012).

Here, we investigated the role of VTA activation in neuronal responses in the rat primary auditory cortex (A1) using *in vivo* intracellular recording. We carefully measured the firing pattern and membrane potential dynamics of A1 neurons and compared the firing rate and EPSP/IPSP (excitatory post-synaptic potential and inhibitory post-synaptic potential) between the control group (sound stimulation only) and the paired stimulation group (paired VTA-sound stimulation). We found that VTA activation can significantly reduce the variability of the sound-evoked response and promote the precision and strength of firing in A1. Furthermore, the compressed response window was caused by an early hyperpolarization resulting from enhanced circuit inhibition.

## MATERIALS AND METHODS

### EXPERIMENTAL PROTOCOL

Animal procedures were approved by the Animal Care and Use Committee of the Third Military Medical University and carried out in accordance with the standards of Care and Use of Laboratory Animals of China. 10 female Sprague-Dawley rats (260–320 g) were anesthetized by Urethane (1.5 g/Kg, intraperitoneal injection). Tracheotomy was performed and animal breathing was kept smooth during the whole experiment. Anesthetized animals were placed above a heating blanket with feedback control to maintain a stable body temperature of 37°C and the animal’s head was fixed by a stereotaxic apparatus (Reward 68000, China). The site for VTA stimulation was determined by a rat brain atlas (bregma: -5.04 mm; right lateral: 0.8 mm; depth: 8 mm below the pia) and confirmed by histology after *in vivo* recording. A small hole (diameter: 1.5 mm) was made in the calvarium (bregma: -5.04 mm; lateral: 0.8 mm) by a dental drill and a cannula (length: 6 mm tube +2 mm base, tube OD: 0.48 mm) was vertically implanted and fixed with dental cement. A tungsten electrode for electric stimulation (2 MΩ, WPI, USA) was inserted into the cannula and slowly advanced into the VTA.

After the implantation of the VTA stimulator, a window for recording (3 mm by 2 mm) was opened within the anatomic range of the primary auditory cortex on the right temporal bone (2.7–5.8 mm posterior to bregma and 3.1–5.4 mm ventral to bregma). The dura was carefully removed with a syringe needle (26 gage) and forceps. Warmed (37°C) artificial cerebral spinal fluid (ACSF in mM: NaCl 124, KCl 2.5, NaH_2_PO_4_ 1.2, CaCl_2_ 2, MgCl_2_ 1, NaHCO_3_ 25, Glucose 20) was used to keep the cortical surface warm and moisturized during the entire experiment.

After surgery, the animals were moved into a double-shielded acoustic room for sound stimulation and electrophysiology recording.

### STIMULATION

#### Sound generation and calibration

The speaker used in this study was an ES1 Free Field Electrostatic Speaker driven by an ED1 Electrostatic Speaker Driver manufactured by Tucker-Davis Technologies (TDT Inc, USA). The sound pressure level (SPL) of pure tones and white noise was calibrated using a 1/4" pressure prepolarized condenser microphone system (377A01 microphone +426B03 preamplifier +480E09 signal conditioner, PCB Piezotronics Inc, USA). The signals were sampled at 1 MHz by a high-speed DAQ board PCI-6251 from National Instruments and our customized Labview program was use for calibration.

#### Pure tones

Pure tones (0.5–64 KHz at 0.1 octave intervals, 25 ms duration, 3 ms ramp) at 8 intensities (varying from 0 to 70 dB, 10 dB interval) were randomly delivered during extracellular and intracellular recordings to locate the position of A1.

#### Sound stimulation and electric stimulation in VTA

**FIGURE 1:**
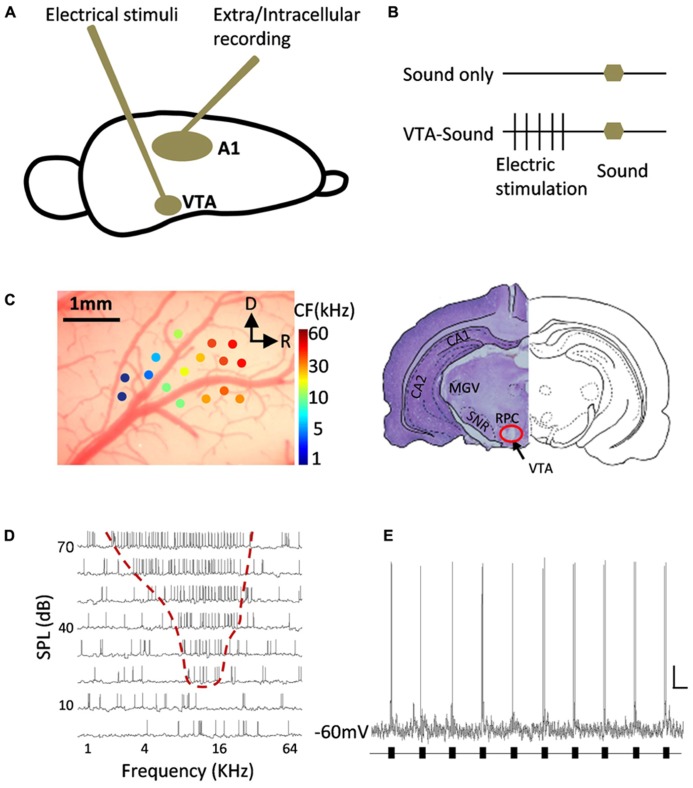
**Diagram of experiment design and example of neuronal responses in A1.** **(A)** A tungsten electrode was inserted into midbrain to electrically stimulate VTA and metal electrodes/sharp glass pipettes were used to record neuronal responses in A1. **(B) **Two groups of stimulation protocols: sound only group (top) and paired VTA-sound group (see Materials and Methods for more details). **(C)** Localization of recording site in A1 and stimulation site in VTA. Extracellular multi-unit recording showed that neurons in A1 have typical gradient in CF from rostral to caudal direction. R, rostral; D, dorsal. The lesion at the stimulation site was well matched with the anatomical position of VTA from the results of cresyl violet staining (right). **(D)** An example of intracellular recording in A1 neuron showed typical “V” shaped frequency-intensity tonal receptive field. **(E)** An example of neuronal responses to repeated sound stimulation (black bars) in A1 recorded by *in vivo* intracellular recording. Scale bar: 10 mV/1s.

Repeated white noise (70 dB, 60 ms duration, 5 ms ramp) was used to test neuronal responses. Electric stimulation was only used in the paired VTA-sound group. The noise began 300 ms after the offset of VTA electric stimulation (monophasic pulses of 0.2 ms duration at 100 Hz and 200 ms train duration, 100 μA). Either sound-only or paired VTA-sound stimulation was repeated more than five times in a random order and the interval between repetitions was at least 6 s (**Figure 1B**).

### ELECTROPHYSIOLOGY RECORDING

#### Extracellular recording

A parylene-coated tungsten electrode (2 MΩ, WPI, USA) was inserted into the cortex and lowered to a depth of 480–650 μm below the pia by a powered manipulator with depth reading (SM-21 Narishige, Japan). A free-field electrostatic speaker was placed 10 cm away from the animal and facing the contralateral ear of the recording site. Pure tones (0.5–64 KHz at 0.1 octave intervals, 25 ms duration, 3 ms ramp) at 8 intensities (varying from 0 to 70 dB, 10 dB interval) were repeated in a pseudorandom order at least five times to measure the receptive field. Recorded signals were amplified and collected by a TDT System 3 (Gain: 5000, sampling rate: 100 KHz, TDT, USA). The high/low pass filter was set at 300/10000 Hz for spiking activities and the threshold for detection was set at three times the standard deviation from the baseline. The multiunit spiking rate and spiking time were automatically analyzed online and recorded for offline analysis by Brainware (TDT Inc., USA). The location of A1 was verified by the typical “V” shaped receptive field, the onset latency (10–20 ms) and the distribution of the characteristic frequency (CF) in the tonotopic map (**Figure 1C**).

#### Intracellular recording

After the identification of A1 with extracellular recording, a sharp glass pipette (filled with 1.0 M potassium acetate, impedance: 80Ω120 Mømega) was inserted into A1 vertically for *in vivo *intracellular recording (400–1200 μm below the pia). Only recordings with a depth of 450 to 650 μm were chosen for this study to ensure the recorded neurons were from layer 4, which is the major recipient layer that receives inputs from the thalamus, according to previous reports (Wang et al., 2006; Sun et al., 2010). The sharp pipette was advanced 2 μm per step from 300 μm below the pia. The intracellular signal was amplified by an Intra767 amplifier (Gain: 100, WPI, USA) and was converted to a digital signal by an analog digital converter micro1401mKII (CED, UK)at a sampling rate of 16667 Hz. The neuron membrane potential was recorded by Spike2 software (CED, UK) and stored for further offline analysis.

#### HISTOLOGY

After the recording phase of each experiment, direct current with an amplitude of 450–550 μA was delivered to the stimulation electrode for 30–50 s to make a lesion at the stimulating site. The rat was deeply anesthetized with an overdose of urethane and perfused intracardially with 4°C pre-cooled 4% paraformaldehyde (PFA) prepared in PB (0.1 M, pH 7.4). The rat brain was quickly removed and dehydrated in 30% sucrose (in 4% PFA), and the brain was sectioned at 18 μm and slices were dried at room temperature. Finally, cresyl violet staining was conducted according to a previously reported method (Lee et al., 2008; **Figure 1C**).

#### DATA ANALYSIS

All off-line analysis, including quantification, plotting, and statistics, were performed using Spike 2 (CED, England), MATLAB (MathWorks, USA), and SYSTAT (Systat Software, USA). For extracellularly recorded data, spiking events were qualified by threshold detection (3 S.D. away from the mean) and averaged across repetitions in 5 ms bins. For intracellular recording, to ensure reliable recording, the resting membrane potential of the recorded neuron had to be lower than -50 mV and had to remain stable during the recording session. Averaged results were presented as the mean ± S.D. unless otherwise noted. The duration of the subthreshold and suprathreshold response was measured by separating the evoked response from spontaneous activity using threshold detection (mean ± S.D.). *T*-tests and paired *t*-tests were used for between-group comparisons. The shape of spike waveforms was used to identify neuron classes according to previous studies (Wu et al., 2008; Moore and Wehr, 2013) and only data from typical excitatory pyramidal cells were chosen for further analysis.

## RESULTS

### VTA ACTIVATION COMPRESSED SPIKING ACTIVITY IN A1 NEURONS

After the implantation of a stimulator in the VTA and the localization of A1, two groups of stimulation protocols were used to test the ability of VTA activation to modulate neuronal activity in A1: sound stimulation only and paired VTA-sound stimulation (**Figures 1A–D**, see Materials and Methods for details). Repeated sound stimulation produced reliable spiking responses measured by *in vivo* intracellular recording (**Figure 1E**).

**FIGURE 2:**
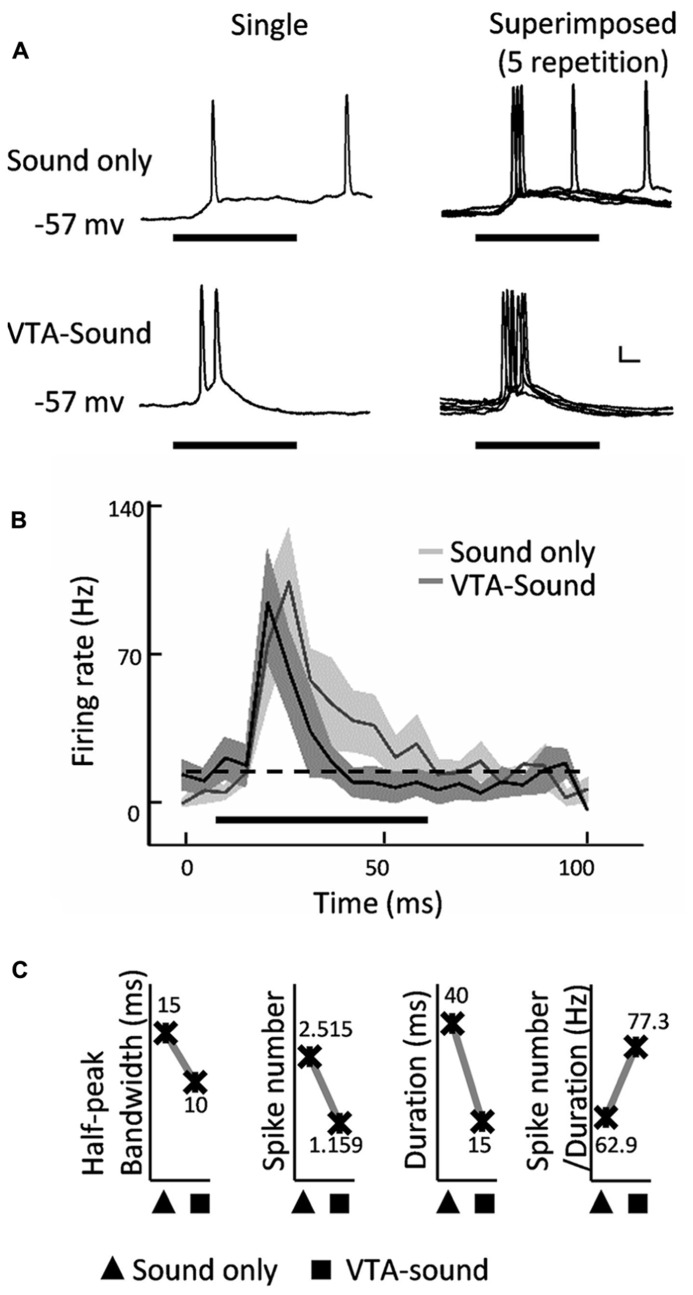
**Spiking response of A1 neurons was compressed by paired VTA-sound stimulation.** **(A)** Example of an intracellular recording from a neuron in rat A1: top, sound only group; bottom, paired VTA-sound group; left, single trace of response; right, five superimposed traces. Thick black bar indicates the sound stimulation. Scale bar: 10 mV/10ms. **(B) **Averaged firing rate (mean ± S.D.) of neuronal responses evoked by two stimulation groups (*n* = 13). Shading indicates the SD. Sound only group: light gray; paired VTA-sound group: dark gray. 5 ms binning window. Dashed line showed the baseline of spontaneous firing. Thick black bar indicates the sound stimulation. **(C)** When VTA-sound stimuli were paired the half-peak bandwidth, spike number and duration of sound evoked spiking responses decreased except that the ratio between spike number and duration increased.

**Figure 2A** shows an example of neuronal responses to different stimulation protocols (sound only and paired VTA-sound) in the primary auditory cortex. Both single trace and superimposed traces from five repetitions are presented. The duration of spiking activity was clearly shortened when VTA and sound stimuli were paired. **Figure 2B** compares the averaged firing rate curves (mean ± S.D. 5 ms bin, 100 ms window) in the different stimulation conditions from 13 intracellularly recorded neurons. When the VTA was activated, the firing rate curve became sharper. To measure the sharpness of the firing rate curve, the half-peak bandwidth was measured as the delay (in ms) between two temporal points (one rising and one falling) where the amplitude of the neural response reached 50% of the maximum level. **Figure 2C** shows that the half-peak bandwidth decreased from 15 to 8 ms when paired VTA-sound stimuli were delivered. The onset latency of the first spike response from each repetition was measured, and no significant difference was found between the two groups (sound only: 15.1 ± 3.3 ms; paired VTA-sound: 14.8 ± 3 ms; *p *= 0.43, paired *t*-test). Meanwhile, when the VTA was activated, the peak amplitude of the firing rate remained similar (sound only: 124.6 Hz; paired VTA-sound: 113.1 Hz) and the latency of the response peak was slightly shorter compared with sound-only stimulation. Considering that the onset latency was very similar and the latency of the peak response between two groups was slightly different, this indicated that the sharpening of the firing rate curve was actually achieved through the shortening of the falling phase of the firing rate curve.

To better characterize the difference between the two firing rate curves, we further compared the duration and total number of spikes in the response window (**Figure 2C**). When sound stimulation was delivered alone, the average number of spikes was 2.515, and duration of the spiking response was 40 ms. When VTA and sound stimulation was paired, the total number of spikes was reduced to 1.159, and the duration of the excitatory response was decreased to 15 ms. These results suggested that VTA activation suppressed the total firing activity in A1 neurons.

Interestingly, while both the number of spikes and the duration of spiking decreased when VTA-sound stimulation was paired, the ratio of spike number to duration was increased by 23% (**Figure 2C**). The ratio was equivalent to the measurement of effective firing density in the corresponding response window. This increase in firing density suggested that although the total number of spikes decreased and the window shortened, the firing strength increased when the VTA was activated by paired electric stimulation.

### SHORTENED EPSPs CAUSED BY VTA ACTIVATION

**FIGURE 3:**
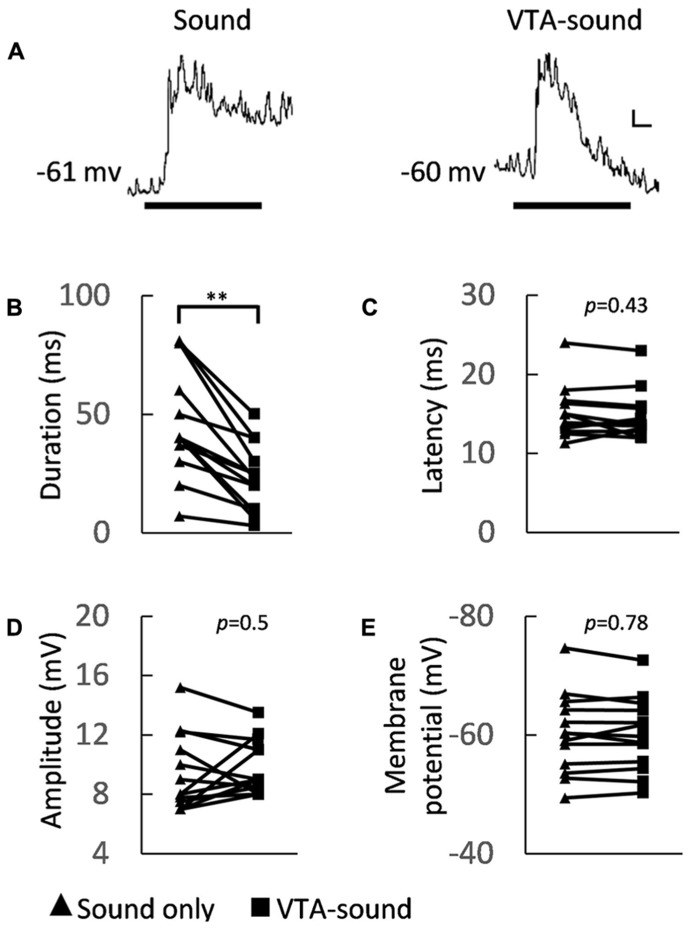
**Membrane potential characteristics of A1 neurons under two groups of stimulation.** **(A)** Averaged membrane potential from 13 recorded neurons (left: sound only group; right: paired VTA-sound group). Thick black bar indicates the sound stimulation. Scale bar: 1 mV/10ms. **(B)** The duration of sound-evoked EPSP became shorter when VTA was activated (sound only: 46.5 ± 23.1 ms; paired VTA-sound: 23.2 ± 14.2 ms, **p  < 0.01, paired *t*-test, *n* = 13). **(C) **The peak latency showed no significant difference between two stimulation groups (sound only: 15.1 ± 3.3 ms; paired VTA-sound: 14.8 ± 3 ms, *p *= 0.43, paired *t*-test, *n* = 13). **(D) **The amplitude of evoked EPSP (sound only: 9.4 ± 2.6 mV; paired VTA-sound: 9.8 ± 1.8 mV, *p *= 0.5, paired *t*-test, *n* = 13) also did not change significantly. **(E)** The membrane potentials before and after the electric stimulation were measured and no significant difference was found (200 ms window; before: -60.2 ± 6.8 mV; after: -60.1 ± 6.2 mV, *p *= 0.78, paired *t*-test, *n* = 13; filled triangle: sound-only stimulation; filled square: paired VTA-sound stimulation).

To obtain a better understanding of the cellular mechanism behind the changes in firing precision and strength, we further analyzed the dynamics of membrane potential changes in A1 neurons. **Figure 3A** shows the averaged membrane potential responses of 13 neurons in A1to sound only and to paired VTA-sound stimulation. The duration of the EPSPs shortened when VTA and sound stimuli were paired. This agreed with previous findings concerning spiking activity (**Figures 2A,B**).

Several indices that can illustrate the membrane potential characteristics of A1 neurons were measured. We found that the amplitude of sound evoked EPSPs and the latency of the first spiking response did not change significantly (sound only/paired VTA-sound: EPSP amplitudes 9.4 ± 2.6/9.8 ± 1.8 mV, *p *= 0.5; latency 15.1 ± 3.3/14.8 ± 3 ms, *p *= 0.43, paired *t*-test, *n* = 13, **Figures 3C,D**), but the EPSP duration was shorter in the paired group (sound only/paired VTA-sound: 46.5 ± 23.1/23.2 ± 14.2 ms, *p * < 0.01, paired *t*-test, *n* = 13, **Figure 3B**). The shortened EPSP duration can explain the suppression in spiking activity caused by VTA activation.

To verify if VTA activation can directly influence the resting membrane potential of A1 neurons, we compared the averaged resting membrane potential before and after electrical stimulation. The results showed no significant changes between the two groups (**Figure 3E**, 200 ms window) before (-60.2 ± 6.8 mV) and after (-60.1 ± 6.2 mV) electrical stimulation (*p *= 0.78, paired *t*-test, *n* = 13). These results indicated that VTA activation does not directly influence the excitability of the recorded neurons in A1, but modulates the neurons through circuit inhibition.

**FIGURE 4:**
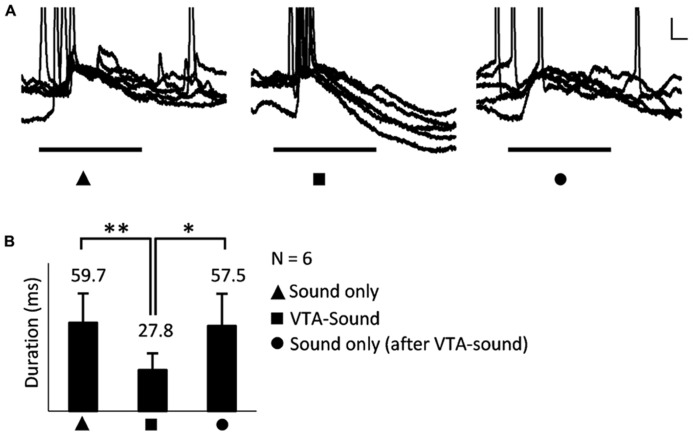
**The shortened EPSP was caused by VTA activation rather than the adaptation to repeated sound simulation.** **(A) **An example of neuronal responses to three stimulation groups: sound only, paired VTA-sound, sound only (again, after paired VTA-sound). Superimposed traces of 5 repetitions. Scale bar: 5 mv/10ms. **(B)** When sound-only stimulation were delivered again the duration of evoked response in A1 neuron was not significantly different from the duration when sound-only stimulation was first applied (*p  < 0.05; **p  < 0.01, paired *t*-test; filled triangle: sound-only stimulation; filled square: paired VTA-sound stimulation; filled circle: sound-only stimulation that was delivered again after paired VTA-sound stimulation).

Adaptation to repeated stimulation has been widely observed and reported in the sensory system (Kilgard and Merzenich, 1998; Chowdhury and Suga, 2000; Kisley and Gerstein, 2001). To ensure that the differences observed here were generated by VTA activation rather than adaptation to repeated sound stimulation, a sound-only protocol was applied again after paired VTA-sound stimulation for a smaller group of neurons (*n* = 6, 15–30 s, random interval, **Figure 4A**). We found that when sound-only stimulation was delivered again, the duration of the evoked response in A1 neurons was not significantly different from the duration when sound-only stimulation was applied for the first time (**Figure 4B**). This result suggested that the early hyperpolarization was caused by VTA activation instead of adaptation to repeated sound simulation. This result also indicated that the influence of VTA activation on auditory neurons in A1 only lasted for a short time.

### VTA ACTIVATION AFFECTS NEURONAL ACTIVITY IN A1 THROUGH CIRCUIT INHIBITION

**FIGURE 5:**
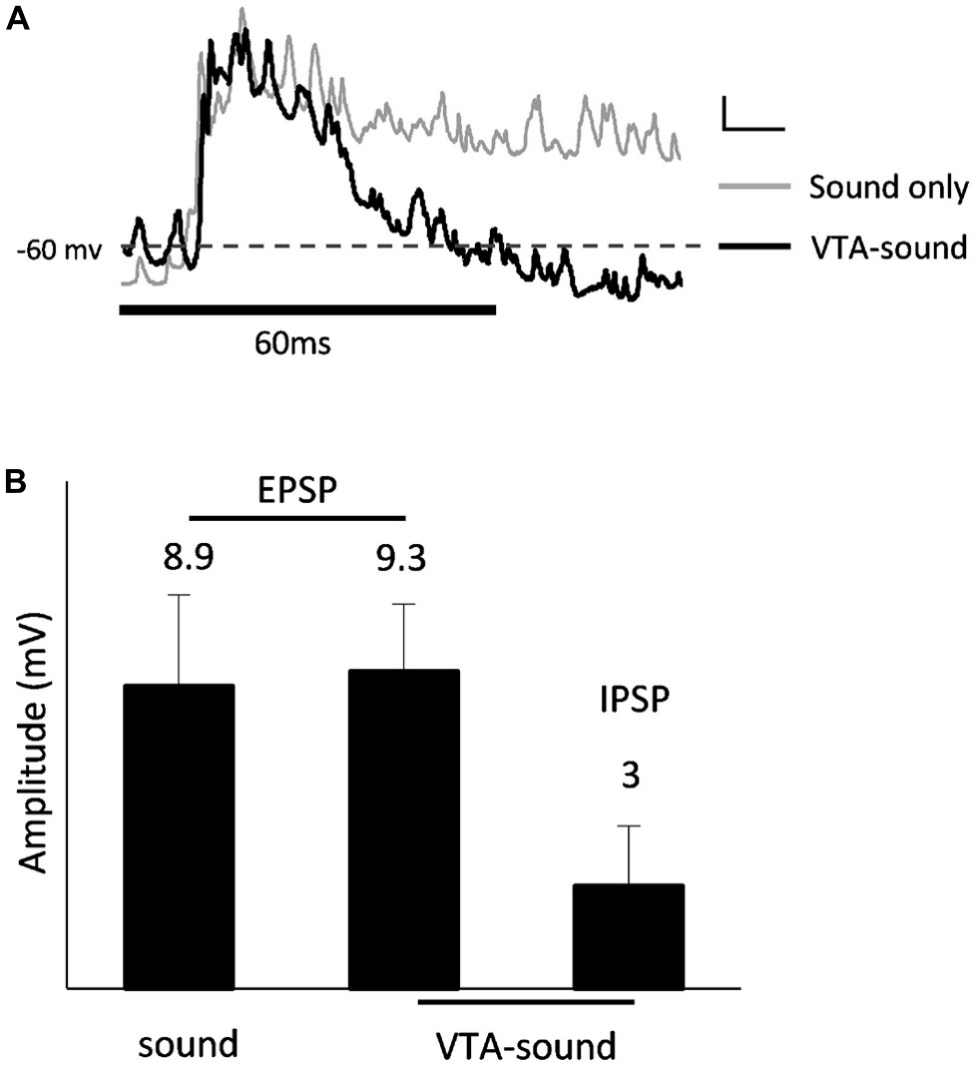
**IPSP after excitatory auditory response.** **(A)** Averaged membrane potential from 13 recorded neurons (sound only group: gray; paired VTA-sound group: black). Thick black bar indicates the sound stimulation. Dashed line shows the level of averaged resting membrane potential. Scale bar: 1 mv/10ms. **(B) **8 of 13 neurons showed clear hyperpolarization after initial depolarization. The amplitudes of EPSP evoked by two stimulation groups showed no significant difference (sound only: 8.9 ± 2.7 ms; paired VTA-sound: 9.3 ± 2 ms, *p *= 0.46, paired *t*-test). Averaged amplitude of IPSP: 3.0 ± 1.8 mV.

When paired VTA-sound stimulation was given, the majority of A1 neurons (8 of 13) showed a clear hyperpolarization approximately 50 ms after the onset of sound stimulation (**Figure 5A**). The amplitudes of the EPSPs evoked in the two stimulation groups showed no significant differences in the 8 neurons (sound only: 8.9 ± 2.7 mV; paired VTA-sound: 9.3 ± 2 mV, *p *= 0.46, paired *t*-test), while the averaged amplitude of IPSPs was 3 ± 1.8 mV (**Figure 5B**). Considering that the peak amplitude of evoked EPSPs in our experiments was approximately 9 mV, these results suggested that the inhibitory modulation caused by VTA activation was strong. As our previous results showed that VTA activation may not directly influence the excitability of recorded neurons in A1, the current results suggest that circuit inhibition accounted for the inhibitory modulation caused by VTA activation.

## DISCUSSION

### THALAMOCORTICAL AND NON-THALAMOCORTICAL PATHWAYS IN A1

Excitatory thalamic inputs can directly activate pyramidal neurons (PNs) or indirectly modulate PNs through GABAergic interneurons in the sensory cortex, which is known as the thalamocortical pathway. The activity of cortical neurons is determined by the integration of thalamic excitatory inputs, intracortical excitation and feedforward inhibition (Ojima and Murakami, 2002; Sun et al., 2010; Han et al., 2012). This circuitry model has been verified in various sensory cortices and is considered to be the basic pathway underlying the response properties of cortical neurons in sensory systems (Douglas and Martin, 1991, 2004; Liu et al., 2007; Zhou et al., 2010; Constantinople and Bruno, 2013).

Aside from the classic thalamocortical pathway, cortical neurons may also receive modulation from other sources, such as the nucleus basalis and VTA. Previous studies have suggested that cholinergic and dopaminergic inputs can modulate neuronal activity and play important roles in learning and plasticity in the auditory cortex (Kilgard and Merzenich, 1998; Dimyan and Weinberger, 1999; Bao et al., 2001; Xiong et al., 2009). Meanwhile it’s also found that orbitofrontal cortex (OFC) can largely mediate cortical activities in A1 through non-cholinergic mechanisms (Winkowski et al., 2013). Cross-modal interactions could shape neuronal functions in auditory cortex as well. Activation of medial agranular motor cortex (M2) can modulate neuronal activity in auditory cortex (Nelson et al., 2013) and a recent study found that in A1 of adult mice, visual deprivation can potentiate thalamocortical synapses and thus enhance auditory information processing (Petrus et al., 2014). Taken together, our results and these earlier findings suggest that the rodent auditory cortex could be regulated by diverse inputs from different areas in the brain.

### *IN VIVO *INTRACELLULAR RECORDING WAS USED TO INVESTIGATE VTA-CORTEX CIRCUITRY

Extracellular recording has been widely used to study neuronal activity in neuroscience research (Nakahara et al., 2004; Ma and Suga, 2005; Yan and Zhang, 2005). Extracellular recording can provide helpful information about the firing patterns of single or multiple neurons. However, it is very difficult to use extracellular recordings to interpret intracellular details such as membrane potential changes (e.g., depolarization/hyperpolarization). *In vivo* intracellular recording is more technically demanding but can provide more details about membrane potential dynamics and clues for possible circuit mechanisms (Douglas and Martin, 1991; Yu et al., 2004; Zhang et al., 2008). Thus, *in vivo* intracellular recording was applied in this study, allowing us to simultaneously measure both the suprathreshold responses (spiking activity) and subthreshold responses (membrane potential changes) from the same neuron.

### THALAMOCORTICAL INPUTS WERE NOT LARGELY AFFECTED BY VTA ACTIVATION

The onset of a sound-evoked neuronal response in A1 is mainly determined by the thalamocortical pathway, while intracortical excitatory and inhibitory inputs contribute to the later phase of evoked activity. The fact that both the suprathreshold onset response (the onset latency of spiking and the peak amplitude of the spiking rate) and the subthreshold onset response (the latency and amplitude of EPSPs) were very similar between the two groups indicated that VTA activation does not largely affect the thalamocortical inputs.

### VTA ACTIVATION PROMOTES FIRING PRECISION AND STRENGTH IN A1 NEURONS

Biological variability is very common in neuroscience research, and a shortened temporal window for spiking activity can reduce uncertainty and increase firing precision for certain acoustic tasks (Wehr and Zador, 2003; Higley and Contreras, 2006; Paille et al., 2013). Shortened response windows may also allow animals to spend less time processing certain acoustic information and promote the efficiency of information processing in the auditory cortex.

Interestingly, although both the total number of spikes and the spike duration dropped when VTA-sound stimuli were paired, the effective firing density (ratio) was increased by 23%. This indicated that stronger firing occurred when the VTA was activated and suggested that auditory neurons become more sensitive to sound stimulation when it is paired with VTA activation.

### EARLY HYPERPOLARIZATION WAS INDUCED BY ENHANCED CIRCUIT INHIBITION

Both the increased firing precision and strength that we observed were mainly due to the shortened response window: the total number of spikes dropped 53.9% and the duration of the spiking response dropped 62.5%. As mentioned before, this shortened response window could be explained by an early hyperpolarization. The shortened spiking window and early hyperpolarization could be generated by many different mechanisms. One possible mechanism is that VTA activation may reduce the excitability of cortical neurons; however, this is not likely because our results showed that the resting membrane potential was not changed by VTA activation. Another possibility is that the early hyperpolarization may be caused by inhibitory circuit inputs to cortical neurons.

### POSSIBLE CIRCUIT MECHANISM UNDERLYING ENHANCED INHIBITION

Our study suggested a possible mechanism of how the reward system affects information processing in the sensory cortex: VTA activation strengthens cortical inhibition, which shortens the response window of post-synaptic cortical neurons and further promotes the precision and strength of neuronal activity.

Recent studies have found that inhibitory circuit inputs are critical to various cortical functions (Gao et al., 2000; Letzkus et al., 2011; Lee et al., 2012; Wilson et al., 2012; Hamilton et al., 2013). Intracortical inhibitory inputs are a major source of circuit inhibition in sensory cortices (Tan et al., 2004; Fino et al., 2013). It is possible that intracortical inhibitory interneurons were responsible for the early hyperpolarization elicited by VTA activation. In rat cortex, parvalbumin (PV), somatostatin (SOM) and vasointestinal peptide (VIP) positive interneurons are the three major subtypes of interneurons that express GABA (γ-aminobutyric acid; Xu et al., 2010). PV+ and SOM+ interneurons may provide inhibitory inputs to excitatory PNs, while VIP+ interneurons are more likely to innervate other inhibitory interneurons (Wilson et al., 2012; Pi et al., 2013). It is likely that VTA activation strengthens the activity of GABAergic interneurons and leads to enhanced circuit inhibition in A1. Further investigation is needed to reveal the interaction between VTA and inhibitory neural networks in rat A1.

## Conflict of Interest Statement

The authors declare that the research was conducted in the absence of any commercial or financial relationships that could be construed as a potential conflict of interest.
